# Bottom-up synthesis of ultra-small molybdenum disulfide-polyvinylpyrrolidone nanosheets for imaging-guided tumor regression

**DOI:** 10.18632/oncotarget.22477

**Published:** 2017-11-08

**Authors:** Jiulong Zhao, Chunhua Zhou, Mao Li, Jialing Li, Guixiang Li, Dan Ma, Zhaoshen Li, Duowu Zou

**Affiliations:** ^1^ Department of Gastroenterology, Changhai Hospital, Second Military Medical University, Shanghai 200433, China; ^2^ Department of Gastroenterology, The Second Affiliated Hospital of Soochow University, Suzhou 215004, China

**Keywords:** molybdenum disulfide, ultra-small, bottom-up, cell nucleus, photothermal therapy

## Abstract

The two-dimensional molybdenum disulfide (MoS_2_) nanosheet has been extensively studied as a novel photothermal transducing agent. However, top-down exfoliation to produce MoS_2_ nanosheets is inefficient, and MoS_2_ nanosheet surface modification procedures are complex. Here, we report the synchronous synthesis and surface modification of 2D MoS_2_ nanosheets with a polyvinylpyrrolidone (PVP)-assisted one-pot hydrothermal method. Due to the chelating-coordinating effect between the lone-pair electrons of the PVP carbonyl oxygen and the unoccupied 4d orbitals of molybdenum, the PVP chains could graft onto the surface of MoS_2_ and guide the growth of the nanosheets. The resultant MoS_2_-PVP nanosheets were ultra-small (21.4 ± 4.4 nm) and exhibited excellent colloidal stability. Moreover, the strong near-infrared absorption of the MoS_2_-PVP nanosheets enabled sensitive photothermal conversion performance (with a mass extinction coefficient of 23.33 L g^−1^ cm^−1^) and *in vitro*/*in vivo* photoacoustic imaging. The MoS_2_-PVP nanosheets had excellent *in vitro* and *in vivo* compatibility and were used for highly efficient tumor photothermal therapy in xenograft tumor-bearing mice. The findings in this report will facilitate the rational design of stable colloidal 2D transition-metal dichalcogenides for effective photothermal cancer therapy.

## INTRODUCTION

Nanomaterials have gained tremendous popularity in the field of biomedicine due to their fascinating physical/chemical properties [1]. The successful exfoliation of graphene sheets from bulk graphite in 2004 by Novosolev, Geim and colleagues initiated the exploration of various kinds of two-dimensional (2D) nanomaterials [2, 3]. Unlike zero- and one-dimensional nanostructures, these novel 2D nanomaterials (including nanosheets, nanoplates and nanodisks) can be one atomic layer thick, which enables greatly compelling quantum size effects and unusual physical properties [4-8]. Due to these advantages, tremendous progress in 2D nanomaterials has been made in the sensor, catalysis, energy storage/conversion and biomedical fields [9-12]. As a typical member of the 2D nanomaterial family, MoS_2_ has been the focus of increasing research interest in the biomedical field [13-17]. Beyond the general properties of biomaterials such as biocompatibility, hemocompatibility and histocompatibility, another notable characteristic of MoS_2_ nanosheets is their strong near-infrared (NIR) absorbance, which enables superior photothermal transformation and photoacoustic imaging for tumor diagnosis and therapy [16, 17].

MoS_2_ has a hexagonal layer structure composed of Mo atoms sandwiched between two layers of S atoms [18, 19]. Since bulk MoS_2_ is stacked layer-by-layer under a weak Van der Waals force, MoS_2_ nanosheets can be “top-down” exfoliated or peeled from the bulk form by means of intercalation reagents or external mechanical force [13, 16, 20-22]. In a pioneer study, Chou et al. synthesized MoS_2_ nanosheets by the top-down chemical exfoliation approach, and this MoS_2_ nanosheet category exhibited a marked photothermal performance improvement over graphene oxide and gold nanorods [13]. Liu et al. reported the possibility of using top-down chemically exfoliated PEGylated MoS_2_ nanosheets as a drug delivery system for synergistic photothermal therapy (PTT) and chemotherapy [16]. Recently, Zhao and co-workers proposed an “improved intercalation-exfoliation process” based on liquid-phase oleum exfoliation for top-down MoS_2_ production. The resultant MoS_2_ nanosheets decorated with chitosan could be used as chemotherapeutic drug nanocarriers for photothermally triggered drug delivery [22].

The top-down approach has been the most frequently studied method of producing MoS_2_ in the laboratory; however, the design of 2D nanomaterials by the top-down method, especially chemical exfoliation, has a series of drawbacks [13, 17]. For instance, the product yield is low, the morphology is difficult to control, and on most occasions, complicated surface modifications are needed [23]. On these grounds, a method of obtaining a biocompatible nanoplatform using only a one-step reaction while simultaneously modifying the surface is desirable to promote the application of MoS_2_ in biomedicine. Shi and colleagues previously proposed a one-pot “bottom-up” solvothermal route for the successful synthesis and surface modification of MoS_2_ nanosheets through solvothermal treatment of an aqueous solution of ammonium tetrathiomolybdate ((NH_4_)_2_MoS_4_) and polyethylene glycol(PEG-400). This bottom-up strategy broke through the bottlenecks of top-down materials synthesis by allowing one-step morphology control and surface modification [17].

Polyvinylpyrrolidone (PVP) is a surfactant that not only controls the particle size, but also imparts colloidal stability to the yielded products [15]. In this study, we extended the one-pot approach to the synthesis of PVP-decorated MoS_2_ nanosheets by selecting ammonium tetrathiomolybdate as the precursor and PVP as the modifier. Due to the chelating-coordinating effect between the lone-pair electrons of the PVP carbonyl oxygen and the unoccupied 4d orbitals of Mo, the PVP chains could graft onto the surface of MoS_2_ and guide the growth of the nanosheets during the hydrothermal process. The ultra-small MoS_2_-PVP nanosheets had strong colloidal stability, high *in vitro* and *in vivo* compatibility and excellent photothermal conversion performance, and were successfully prepared and used for highly efficient tumor PTT in a xenograft tumor-bearing mouse. Due to their NIR absorbance, these ultra-small MoS_2_ nanosheets were sensitive *in vitro* and *in vivo* photoacoustic imaging contrast agents. To our knowledge, this is the first study concerning the synthesis and morphology control of ultra-small MoS_2_ nanosheets and their use in tumor PTT therapy. This research highlights the possibility of surface modification based on the chelating-coordinating effect between polymer chains and the unoccupied orbitals of transition metal atoms.

## RESULTS AND DISCUSSION

**Figure 1:**
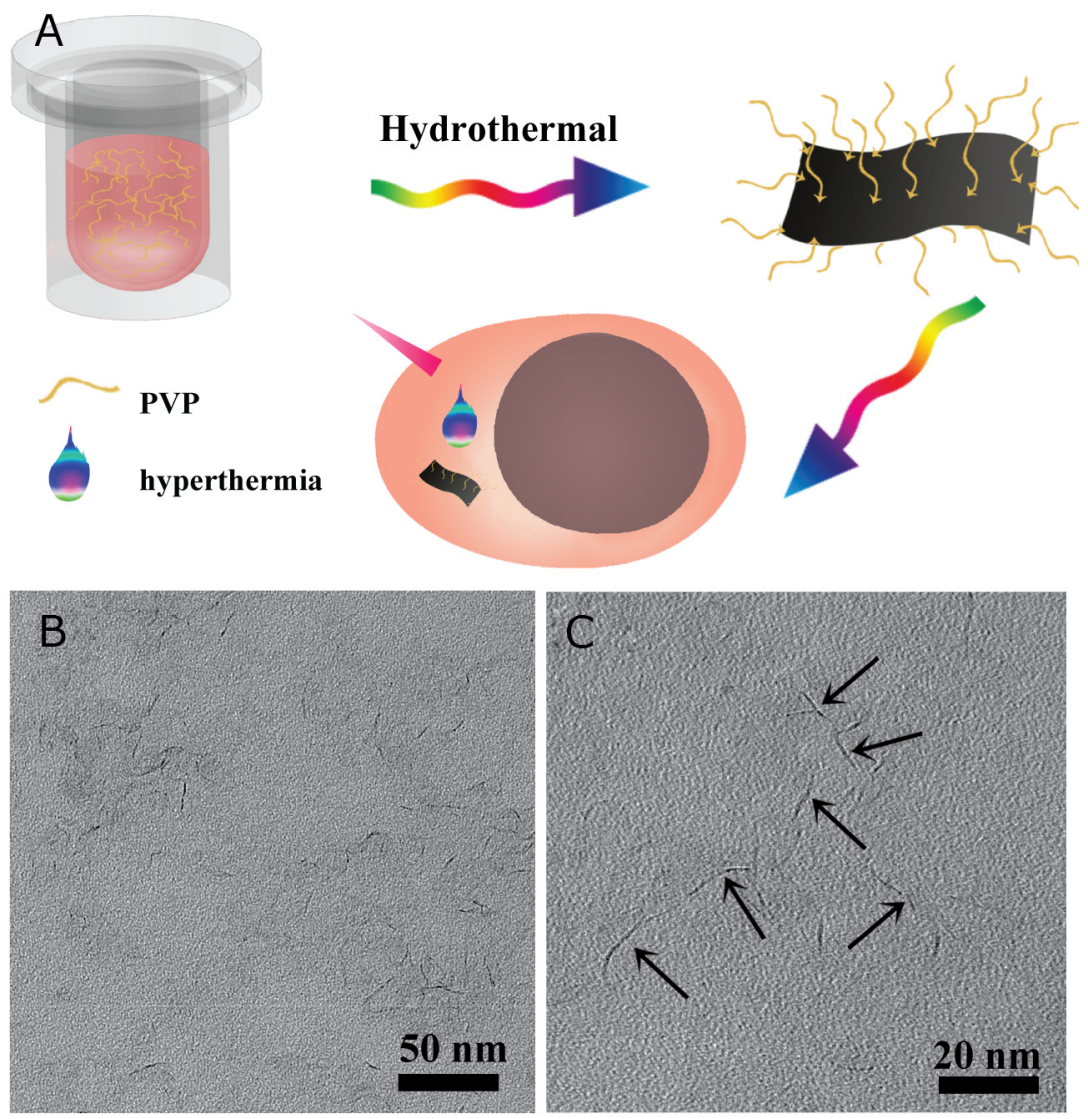
**(A)** Schematic illustration of the solvothermal synthesis procedure of MoS_2_-PVP nanosheets and PTT; **(B)** TEM image of MoS_2_-PVP nanosheets; **(C)** enlarged TEM image of (B).

The overall design concept and schematic hydrothermal treatment procedure for the synthesis of MoS_2_-PVP nanosheets can be found in Figure 1A. The lone-pair electrons of the carbonyl oxygen of PVP could chemically react with the empty orbitals of Mo via the chelating-coordinating effect. Due to this molecular-level force, the PVP chains could confine the growth of the nanosheet and guide the material extension along the polymer chains. On the other hand, this chelating-coordinating effect made it possible for the surface PVP to trap the nanomaterials. Without the addition of PVP, the size of the produced sheet-like MoS_2_ was about 50 nm (Supplementary Figure 1). Although atomic force microscopy is a powerful method to determine the thickness of nanosheets, it was unsuitable for the MoS_2_ nanosheets prepared by our “bottom-up” method because their morphology was crooked.

The Mw and concentration of PVP influenced the growth of the nanosheets (Supplementary Figures 2 & 3). When the Mw of the added PVP was 360 kDa, the product retained a well-defined and uniform sheet-like shape, but the size dramatically decreased to 21.4 ± 4.4 nm and the thickness was about 1 nm (0.9 ± 0.2 nm, Figure 1B). PVP with a Mw of 30 kDa guided the growth of the MoS_2_ nanosheets; however, because the polymer chains were short, the weak constraining force was insufficient to support nanosheet growth, so impure-phase nanoparticles were formed (Supplementary Figure 2). We found that even smaller MoS_2_ nanosheets (14.7 ± 1.2 nm) could be synthesized when the PVP concentration was doubled (Mw = 360 kDa, 10 mg/mL, Supplementary Figure 3).

These results clearly indicated that the size and morphology of the nanosheets could be altered through tuning of the PVP Mw and concentration. Although a detailed mechanism has not been clearly established yet, it seems likely that the PVP concentration and the length of the polymer chain influence the chelating-coordinating force and thus induce these size and morphology changes. Considering the well-defined 2D particulate morphology and colloidal stability (discussed below) of PVP with a Mw of 360 kDa, we selected it as the modifier for the one-pot synthesis and surface modification of MoS_2_ nanosheets.

**Figure 2:**
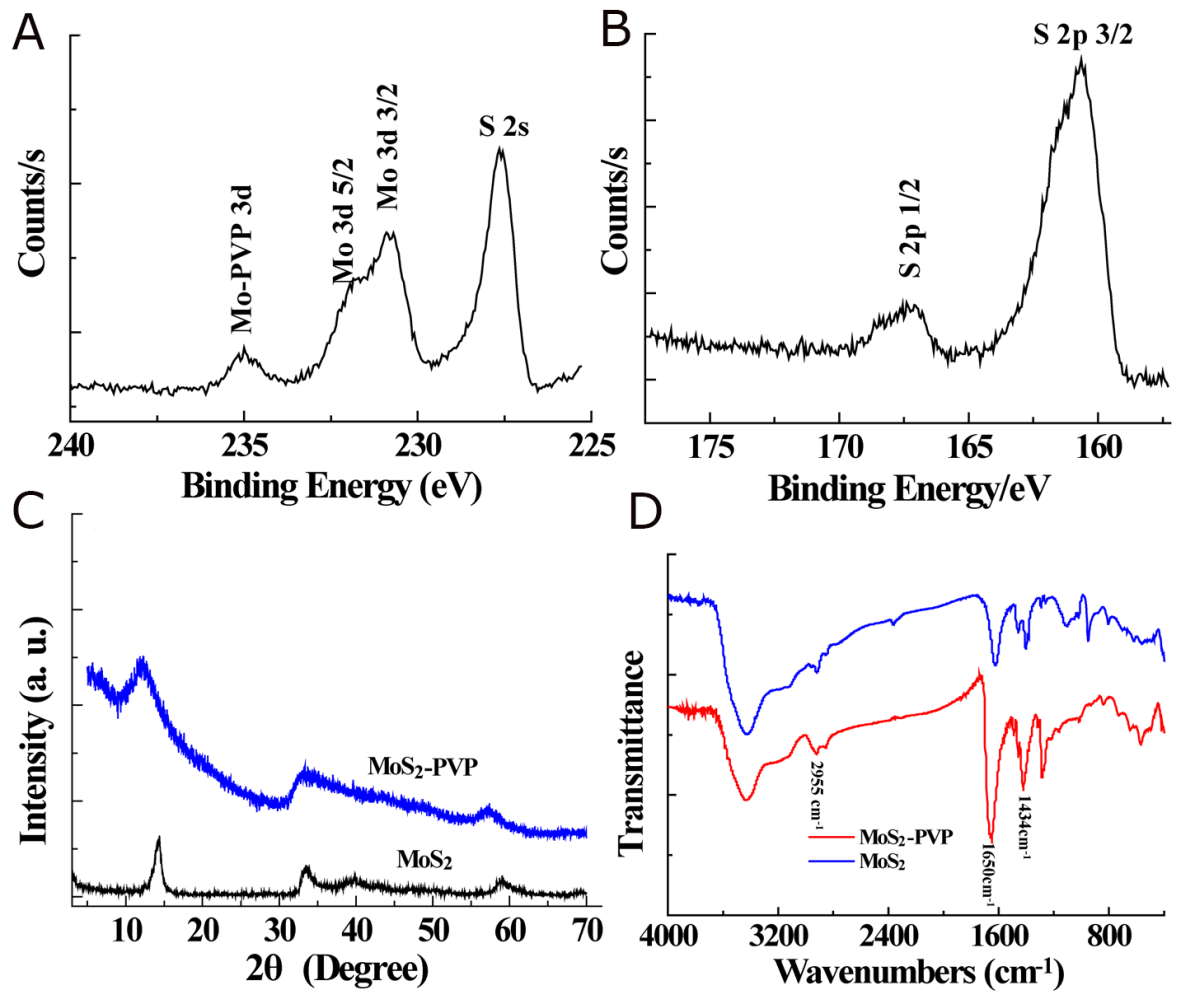
**(A)** XPS spectrum of Mo 3d and S 2s orbits; **(B)** XPS spectrum of S 2p orbits. **(C)** XRD patterns and **(D)** FTIR spectra of pure MoS_2_ and MoS_2_-PVPnanosheets.

XPS and XRD were performed to analyze the chemical composition and structure of the nanosheets (Figure 2A-2C). Characteristic peaks of MoS_2_ located at 229.2 eV (Mo 3d 5/2) and 230.8 eV (Mo 3d 3/2) (corresponding to Mo^4+^), as well as at 227.6 eV (S 2s), 167.4 eV (S 2p 1/2) and 160.1 eV (S 2p 3/2) (corresponding to S^2-^) were detected in the XPS spectra (Figure 2A, 2B). With the exception of C, O and Si, which originated from the surface-modified PVP and the sample scaffold for EDS analysis, no other element was observed in the EDS spectrum (Supplementary Figure 4). Moreover, the XRD pattern matched the crystalline structure of MoS_2_ well (Figure 2C).

Fourier transform infrared spectroscopy was carried out to determine the success of the PVP grafting during the hydrothermal process. A remarkable peak intensity increase was found at 1650 cm^-1^, belonging to the stretching vibration of the carbonyl groups of PVP (Figure 2D). Moreover, two peaks were clearly detected at 2955 cm^-1^ and 1434 cm^-1^, which could be ascribed to the stretching and bending vibrations of C-H bonds, indicating the existence of PVP chains on the nanosheet surface. These characterization data suggested that the chemical essence of the as-prepared nanosheet was MoS_2_, and that the PVP introduced during the synthesis was successfully anchored onto the product surface (noted as MoS_2_-PVP).

**Figure 3:**
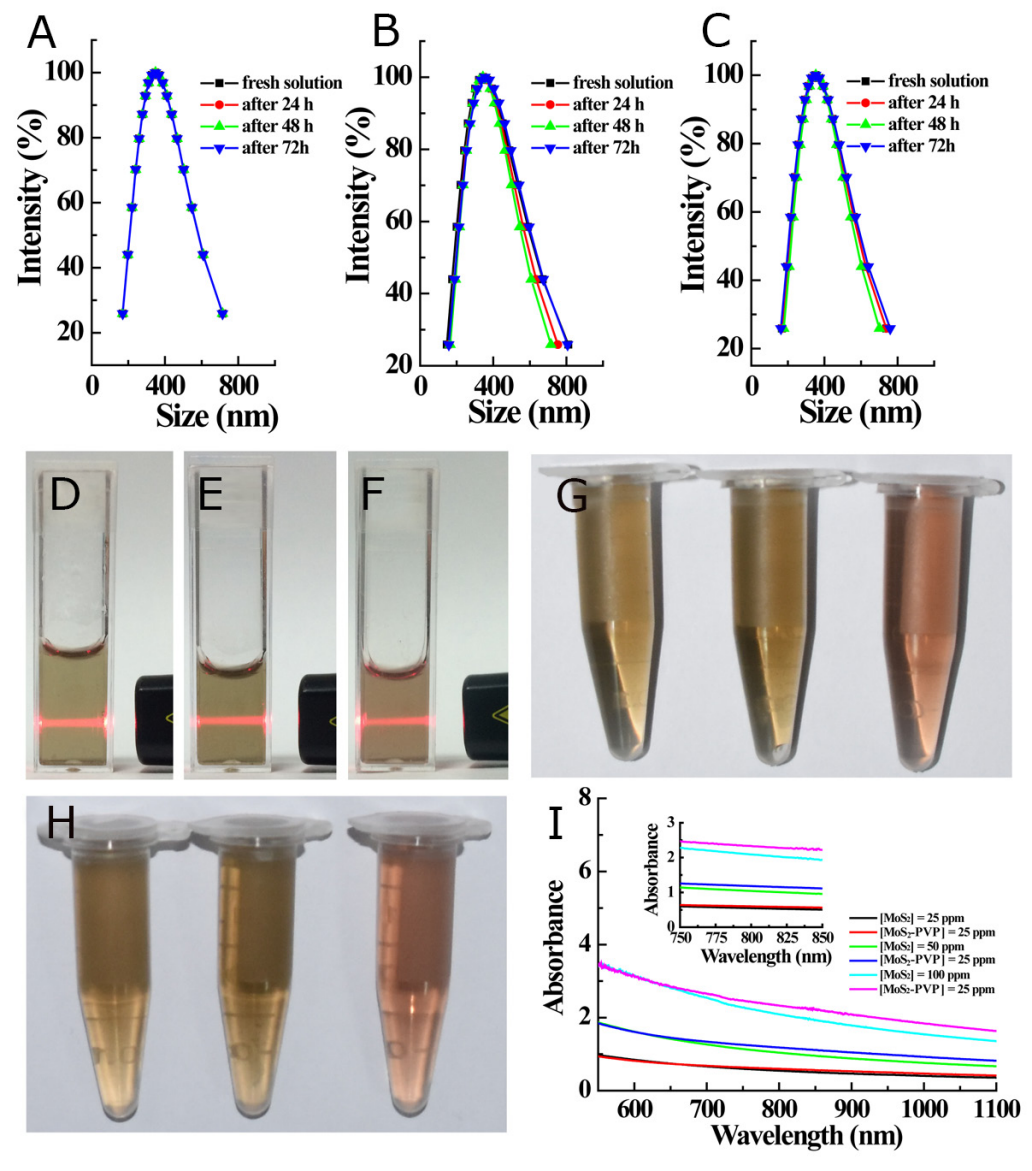
**(A-C)** Time-dependent DLS of MoS_2_-PVP nanosheets in different media: (A) distilled water, (B) saline, and (C) RPMI 1640 cell culture medium; **(D-F)** photographic images of typical Tyndall light scattering of MoS_2_-PVP in different media: (D) distilled water, (E) saline, (F) RPMI-1640 cell culture medium; **(G)** photographic image of fresh MoS_2_-PVP aqueous solutions (left: distilled water, middle: saline, right: RPMI 1640 medium); **(H)** photographic images of MoS2-PVP aqueous solutions (left: distilled water, middle: saline, and right: RPMI 1640 medium) after three days standing; **(I)** UV-Vis-NIR spectra of MoS2 and MoS2-PVP aqueous solutions at different concentrations; the insert contains a zoomed-in picture of the light absorption at 750-850 nm.

The surface-decorating PVP chains guaranteed the excellent colloidal stability of the MoS_2_-PVP nanosheets in different surroundings. They could be well-dispersed in water, saline, RPMI 1640 cell culture medium and FBS to form stable dispersions (Figure 3G & Supplementary Figure 5) with the typical Tyndall effect (Figure 3D-3F). The DLS diameters of MoS_2_-PVP nanosheets in the above dispersions exhibited negligible fluctuations within 72 h standing at room temperature (Figure 3A-3C & 3H). It is worth noting that the DLS diameter was larger than the TEM size (Figure 1B-1C), which may have been due to the formation of a hydration shell around the nanosheets after the anchoring of the surface PVP molecules. Apparently, the surface PVP modification enhanced the colloidal stability much more effectively than PEG-400, which only maintained stability under physiological conditions for 24 h [17].

### *In vitro* photothermal performance

To explore the cancer PTT treatment potential of the as-prepared MoS_2_-PVP nanosheets, we next focused our research efforts on their light-absorption features. Similar to top-down exfoliated 2D MoS_2_ nanosheets [13], MoS_2_-PVP nanosheets exhibited broad absorption in the NIR region (Figure 3G, the insert contains a zoomed-in picture of the light absorption at 750-850 nm). The NIR absorption of nanosheets correlates closely with their size; thus, the ultra-small MoS_2_-PVP nanosheets could absorb more from the NIR laser than large MoS_2_ nanosheets (50 nm) at the same Mo concentration (Figure 3I). The mass extinction coefficient of the MoS_2_-PVP nanosheets at 800 nm was calculated to be 23.33 L g^−1^ cm^−1^, which was much higher than that of graphene oxide (3.6 L g^−1^ cm^−1^) and similar to those of reduced graphene oxide (24.6 L g^−1^ cm^−1^) and chemically exfoliated MoS_2_-PEG nanosheets [16].

**Figure 4:**
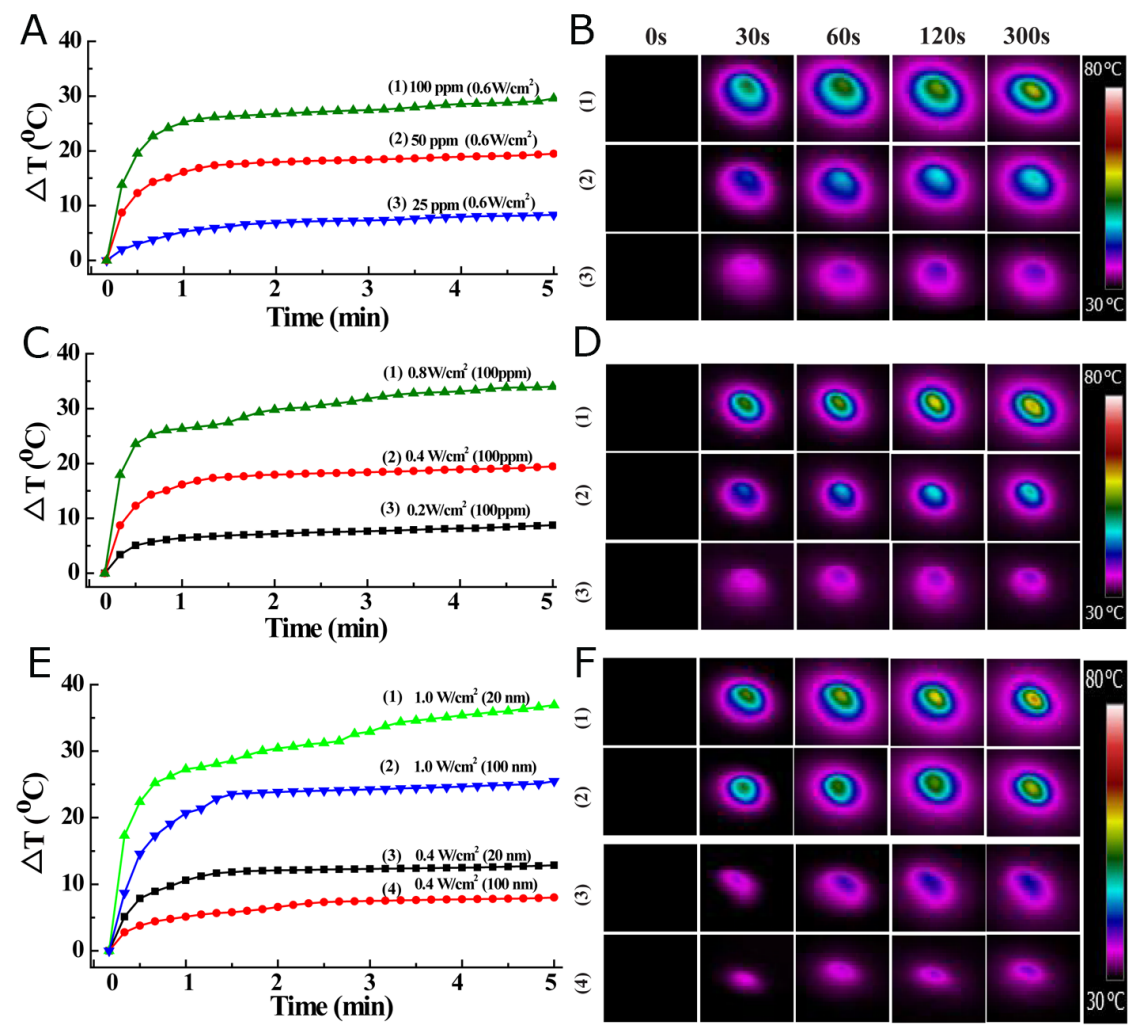
Temperature-change profiles of MoS_2_-PVP aqueous dispersions as a function of **(A)** MoS_2_-PVP concentration (power density: 0. 6 W/cm^2^), **(C)** power density (MoS_2_ concentration: 100 ppm), and **(E)** size (power density: 0.4 or 1.0 W/cm^2^); **(B, D, F)** thermal images of MoS_2_-PVP aqueous solutions, corresponding to panels (A, C, E).

The obvious NIR laser absorbance, along with the excellent hydrophilicity and dispersity in different physiological media, encouraged us to pursue the potential of MoS_2_-PVP nanosheets as photothermal agents. For this purpose, we exposed a series of MoS_2_-PVP nanosheet dispersions to the NIR laser at a pre-determined power density to explore the correlation of the photothermal performance with the concentration, irradiation time and power density. The photothermal conversion capacity of the MoS_2_-PVP nanosheets depended on the nanosheet concentration and power density. The temperature increases (∆T) were 8.3 °C, 19.5 °C and 29.6 °C when the Mo concentrations were 25, 50 and 100 ppm, respectively, at a low power density of 0.6 W/cm^2^ (Figure 4A, 4B). Similarly, the ∆T of the MoS_2_-PVP nanosheet solution increased with the NIR laser power density (Figure 4C, 4D), being 8.9 °C, 19.7 °C and 34.7 °C when the laser power densities were 0.2 W/cm^2^, 0.4 W/cm^2^ and 0.6 W/cm^2^, respectively. Unlike the MoS_2_-PVP nanosheets, pure water exhibited no detectable temperature variation (∆T = 2.1 °C), even at a power density of 1.0 W/cm^2^ (Supplementary Figure 6).

The influence of the surface PVP molecules on the photothermal performance was also investigated. As MoS_2_-PVP nanosheets exhibited stronger laser absorbance than MoS_2_ nanosheets without surface modification (Figure 3I), they also displayed enhanced photothermal performance, with ∆T increases of about 5.3 and 7.2 °C at power densities of 0.4 W/cm^2^ and 1.0 W/cm^2^, respectively (Figure 4E, F). In addition to excellent photothermal performance, the MoS_2_-PVP nanosheets demonstrated desirable photothermal stability. In cycling temperature-change monitoring experiments, the maximum temperature change during five cycles of irradiation displayed no appreciable variation (Supplementary Figure 7, 50 ppm, 0.6 W/cm^2^), implying that five cycles of NIR laser irradiation did not significantly influence the photothermal stability of the MoS_2_-PVP nanosheets. The unique NIR absorbance, excellent photothermal performance and great stability of the MoS_2_-PVP nanosheets highlight their potential as stable and durable photothermal agents for tumor PTT.

### Cellular uptake of MoS_2_ nanosheets

The intracellular uptake of MoS_2_-PVP nanosheets could substantially influence their effectiveness for *in vitro* cancer cell PTT. Tunable cellular uptake is a prerequisite for effective PTT. A large number of MoS_2_-PVP nanosheets that were trapped by small vesicles in the cytoplasm were observed by Bio-TEM (Supplementary Figure 8A, indicated by the arrows), clearly indicating that the MoS_2_ nanosheets were taken up by cells via endocytosis (Supplementary Figure 8B). Further quantitative evaluation by ICP-OES demonstrated that the specific cellular uptake amount was ∼80 μg/well. Similar to surface PEG grafting [25], surface PVP-chain anchoring was anticipated to facilitate nutritional absorption from the cell culture medium and promote the cellular uptake of MoS_2_-PVP nanosheets. We expected that this material uptake by cancer cells, especially the cell nuclei, would greatly enhance the PTT performance of the MoS_2_-PVP nanosheets by enabling them to target and destroy the nucleus passively via hyperthermia under NIR irradiation.

### *In vitro* cytocompatibility and PTT

**Figure 5:**
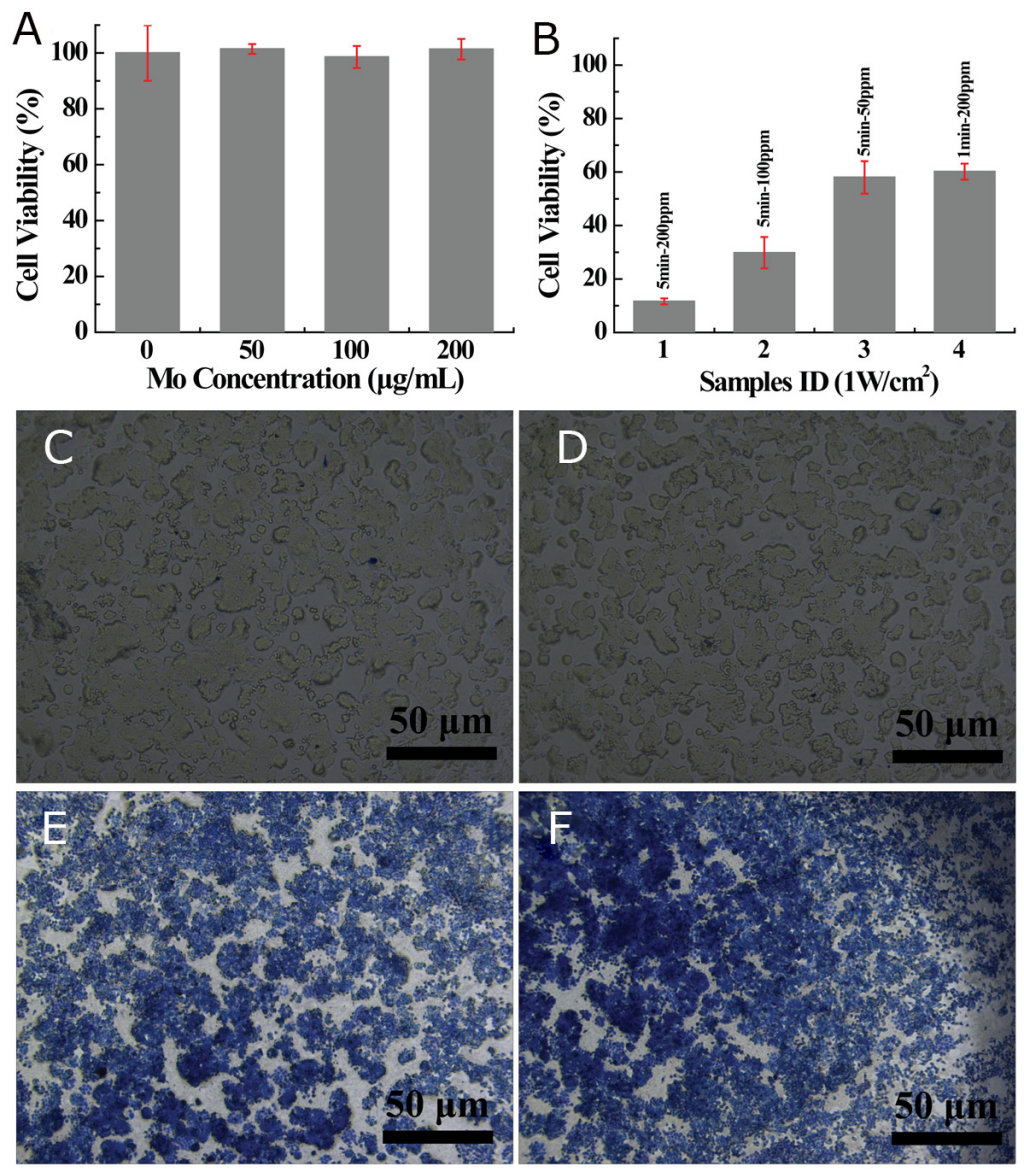
**(A)** Viability of HT29 cells incubated with different concentrations of MoS_2_-PVP nanosheets for 24 h; **(B)** *in vitro* PTT performance (irradiation time: 1 min or 5 min); **(C-F)** phase-contrast images of trypan blue-stained HT29 cell morphology: (C) HT29 cells treated with saline (control), (D) HT29 cells treated with MoS_2_-PVP nanosheets (concentration: 200 μg/mL); (E, F) Cells treated with MoS_2_-PVP nanosheets at a concentration of 200 μg/mL and NIR irradiation for 1 min and 5 min, respectively.

In order to be safely applied in biomedicine, MoS_2_-PVP nanosheets should not cause any cellular damage or toxicity. Therefore, we next evaluated the *in vitro* cytocompatibility of MoS_2_-PVP nanosheets using the standard CCK-8 assay. The MoS_2_-PVP nanosheets were minimally toxic to both HT29 and L929 cells. After these cells were incubated with MoS_2_-PVP nanosheets for 24 h, their viabilities were negligibly reduced, even at a high nanosheet concentration of 200 μg/mL (L929: ∼87% [Supplementary Figure 9] and HT29: ∼100% [Figure 5A]). In addition to the CCK-8 assay, trypan blue staining (which stains apoptotic/dead cells blue) was performed as further proof of the negligible cytotoxicity of the MoS_2_-PVP nanosheets. Similar to the results in saline-treated HT29 (Figure 5C) and L929 cells (Supplementary Figure 9B), the morphological integrity of HT29 and L929 cells was not destroyed after the cells were incubated with MoS_2_-PVP nanosheets at a high concentration of 200 μg/mL (Figure 5D and Supplementary Figure 9C), confirming that the cytotoxicity of MoS_2_-PVP nanosheets was negligible at the experimental dosage (0-200 μg/mL).

After demonstrating the excellent NIR-absorbing capacity and cytocompatibility of the MoS_2_-PVP nanosheets, we then studied their effectiveness for *in vitro* cancer cell photothermal ablation. When HT29 cells were incubated with MoS_2_-PVP nanosheets and irradiated with the NIR laser, their cellular viability decreased concentration-dependently (Figure 5B, bars 1-3). When incubated with MoS_2_-PVP nanosheets at a concentration of 200 ppm for 6 h, the majority of HT29 cells were killed by NIR-induced hyperthermia, such that their viability was reduced to ∼11% (Figure 5B, bar 1). When the nanosheet concentrations were reduced to 100 or 50 ppm, the cellular viability levels were 29.8 ± 5.9% or 58.0 ± 6.1%, respectively (Figure 5B, bars 2 and 3). The apoptosis induced by hyperthermia was so swift that 40% of the cells died within 1 min (200 μg/mL, cell viability of 60.2 ± 3.1%, bar 4).

The *in vitro* cancer cell photothermal ablation was further evaluated with trypan blue staining. Consistent with the CCK-8 results, a large portion of the MoS_2_-PVP (200 μg/mL)-treated HT29 cells were stained blue (Figure 5D) after irradiation with the laser for 1 min, while after 5 min of irradiation, almost all of the HT29 cells were stained blue (Figure 5F). These results suggested that the MoS_2_-PVP nanosheets efficiently converted NIR light into thermal energy and significantly suppressed *in vitro* cell proliferation by inducing hyperthermia.

### *In vivo* compatibility of MoS_2_ nanosheets

The *in vitro* hemocompatibility of the MoS_2_-PVP nanosheets toward mRBCs was evaluated by hemolysis, coagulation, serum biochemistry and routine blood testing [26]. The hemolytic assay was not applicable because the ultra-small MoS_2_-PVP nanosheets (which would have significantly influenced the light absorption) were difficult to separate from the mRBC dispersion, even at a high centrifugal speed of 12000 rpm. Therefore, after the mRBCs were incubated with MoS_2_-PVP nanosheets (200 μg/mL) for 2 h, the cellular morphology was observed by Wright’s staining method. The results (Supplementary Figure 10) clearly indicated that the structural integrity of the mRBCs was well-maintained after the cells were treated with MoS_2_-PVP nanosheets.

When KM mice were I.V. injected with MoS_2_-PVP nanosheets, coagulation functional assessment at days 1 and 28 revealed that the plasma coagulation indexes (APTT, FDP, FIB, PT and TT) remained in the same range as the control values (from saline-treated plasma) (Supplementary Figure 11), indicating that the MoS_2_-PVP nanosheets did not cause the coagulation. To assess the hemocompatibility further, we performed blood biochemistry and routine blood tests on days 1, 14 and 28 after I.V. injection of MoS_2_-PVP nanosheets. No significant physiological differences in various routine blood indexes were found between the experimental and control mice, further confirming the favorable hemocompatibility of MoS_2_-PVP nanosheets at the experimental dosage (Supplementary Figure 12).

**Figure 6:**
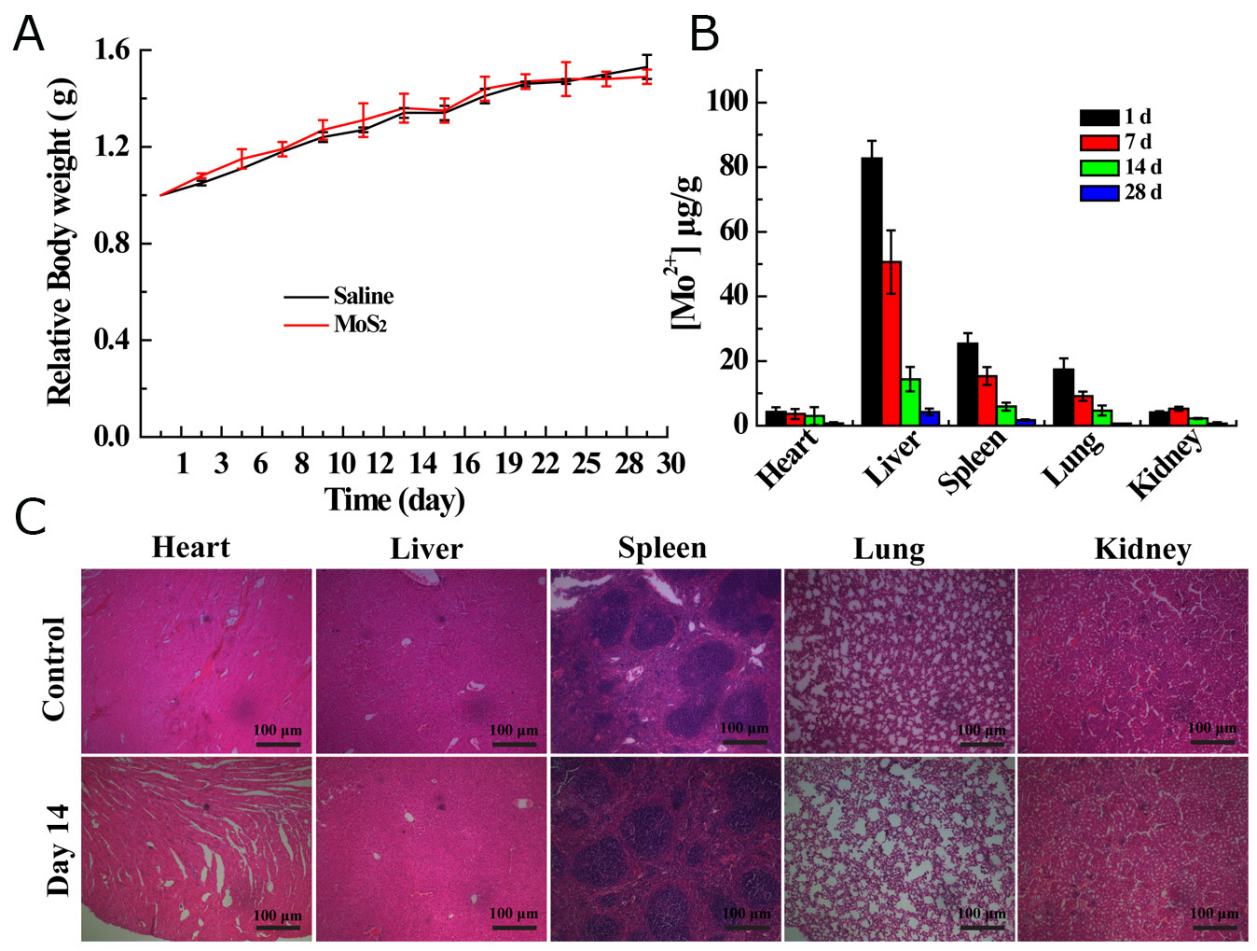
**(A)** Body weight changes of KM mice I. V. injected with saline or MoS_2_-PVP nanosheets; **(B)** biodistribution of Mo in the heart, liver, spleen, lung and kidney at 1, 7, 14 and 28 days after I.V. injection with MoS_2_-PVP nanosheets (mean ± standard deviation, n = 3). **(C)** H&E staining images of hearts, livers, spleens, lungs and kidneys from KM mice on day 14 after treatment with saline (control) or MoS_2_-PVP nanosheets (magnification: 100 ×).

To demonstrate the *in vivo* safety of the MoS_2_-PVP nanosheets, we then monitored the body weights, MoS_2_-PVP nanosheet biodistribution patterns and tissue lesions of KM mice (Figure 6) after MoS_2_-PVP nanosheet administration. KM mice in both the control and treatment groups presented natural body weight increases and exhibited no obvious weight variation over the feeding time (Figure 6A). The amount of Mo in major organs (heart, liver, spleen, lung and kidney) after material administration was determined in KM mice sacrificed on days 1, 7, 14 and 28. High levels of the I.V.-injected materials were observed in the liver and spleen on day 1, indicating their accumulation in the reticuloendothelial system within the first 24 h [27]. Fortunately, the Mo levels in all organs decreased with time, and were quite low after 28 days, suggesting that the nanosheets were excreted from these organs (Figure 6B). The *in vivo* metabolism of the as-synthesized MoS_2_-PVP nanosheets was similar to that previously reported for MoS_2_-PEG nanosheets [17], possibly indicating that the surface-decorating PVP macromolecules improved the blood circulation duration of the MoS_2_ nanosheets. It was difficult to determine the biodistribution of the pure MoS_2_ nanosheets, due to their poor colloidal stability.

To investigate whether the organ accumulation of the MoS_2_-PVP nanosheets would exert any adverse effects, we observed the tissue lesions of these organs with H&E staining. After MoS_2_ nanosheet administration and subsequent four-week feeding, no signs of acute or chronic pathological toxicity or adverse effects were observed in the control or treatment groups (Figure 6C). In general, the surface PVP-decorated MoS_2_ nanosheets exhibited excellent *in vivo* hemo/histocompatibility, and demonstrated promising potential as nano-agents for the PTT treatment of cancer.

### *In vitro* and *in vivo* photoacoustic imaging

**Figure 7:**
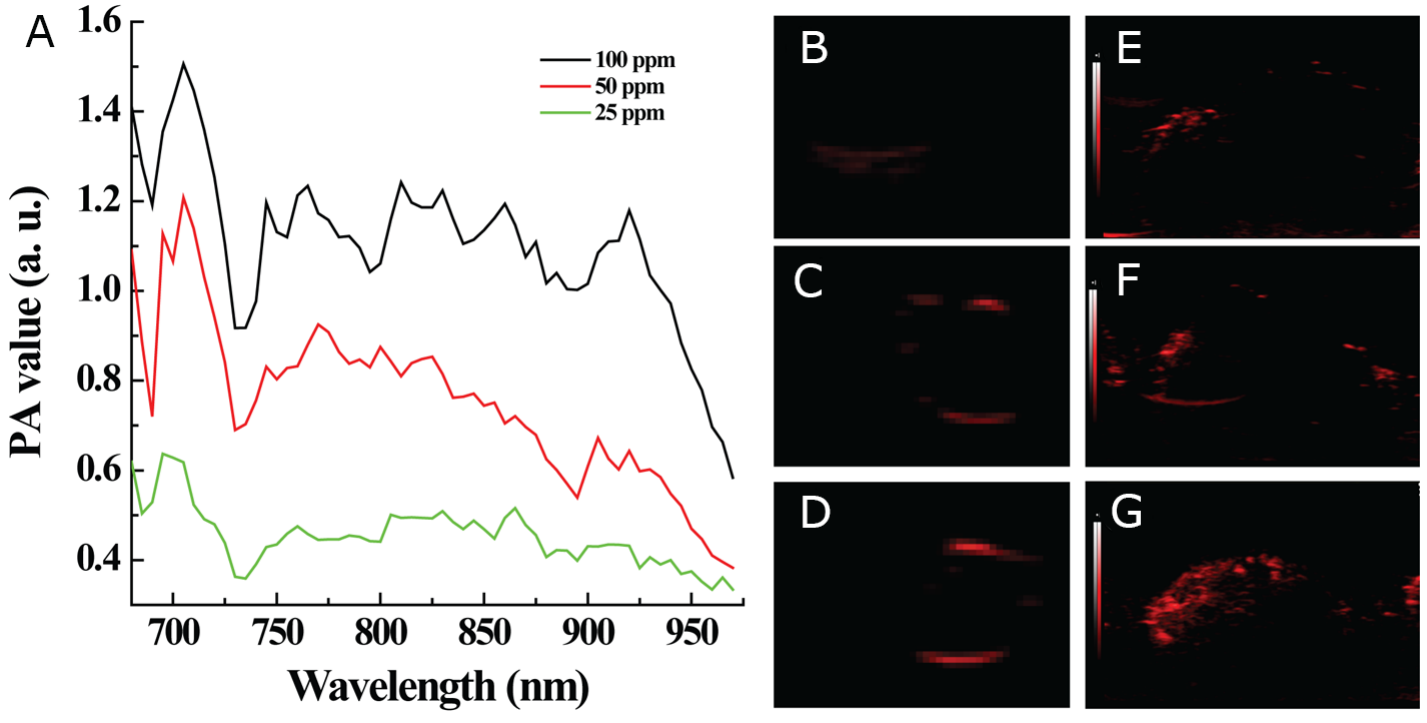
*In vitro* and *in vivo* photoacoustic imaging **(A)** *In vitro* photoacoustic (PA) values versus MoS_2_-PVP nanosheet concentrations within the laser wavelength of 690-970 nm; **(B-D)** photoacoustic images at MoS_2_ concentrations of 25, 50 and 100 ppm, respectively, under NIR irradiation (808 nm); **(E-G)** *in vivo* photoacoustic images of tumors obtained from (E) a control mouse, (F) a mouse I.V. injected with MoS_2_-PVP nanosheets and (G) a mouse I.T. injected with MoS_2_-PVP nanosheets, under NIR irradiation (808 nm).

An ideal nanoscale NIR laser-absorber for photoacoustic imaging not only would have a sufficiently high extinction coefficient in the NIR region, but also would exert NIR irradiation-induced photoacoustic effects [28-30]. Therefore, MoS_2_-PVP nanosheets, which have outstanding NIR laser absorbance, should also have great potential as photoacoustic imaging contrast agents. To verify this hypothesis, we dropped different concentrations of MoS_2_-PVP nanosheets into small holes in a home-made agar plate, and scanned them to record their photoacoustic effects under NIR irradiation (680-970 nm). Figure 7A presents the photoacoustic signal intensities of MoS_2_-PVP nanosheet aqueous dispersions at different concentrations. The photoacoustic effect increased with the material concentration, as evidenced by the gradual deepening in brightness with increasing Mo concentrations (25 to 100 ppm). Moreover, the contrast could be clearly visualized even at a low Mo concentration of 25 ppm (Figure 7B).

This superior *in vitro* photoacoustic imaging capacity motivated us to assess the *in vivo* tumor imaging performance of the MoS_2_-PVP nanosheets in tumor-bearing Balb/c nude mice. NIR lasers with wavelengths of 808 nm and 980 nm are two frequently used laser sources in tumor PTT. Since the absorbance of WS_2_-PVP nanosheets was higher at 808 nm than at 980 nm, we chose 808 nm as the laser wavelength for quantitative photoacoustic imaging [8]. At a wavelength of 808 nm, the photoacoustic signal intensity increased dramatically in HT29-xenografted tumors, regardless of whether I.T. or I.V. material injection was performed (Figure 7E-7G), confirming the possibility of using ultra-small MoS_2_-PVP nanosheets as qualified photoacoustic contrast agents *in vivo*.

### *In vivo* PTT performance of MoS_2_-PVP nanosheets

Given the biocompatibility, tumor imaging capacity and *in vitro* tumor PTT efficiency of MoS_2_**-**PVP nanosheets, we then carried out animal experiments to determine their *in vivo* tumor PTT effectiveness. Tumor-bearing Balb/c nude mice were randomly divided into three groups, to be either I.V. injected with saline (control), I.V. injected with MoS_2_-PVP nanosheets, or I.T. injected with MoS_2_-PVP nanosheets. Then, the mice in the control and I.T.-injected groups were immediately exposed to the NIR laser for 5 min, while the mice in the I.V. material injection group received the 5-min laser irradiation 12 h after the injection, to allow the MoS_2_**-**PVP nanosheets to permeate the tumor tissue thoroughly.

**Figure 8:**
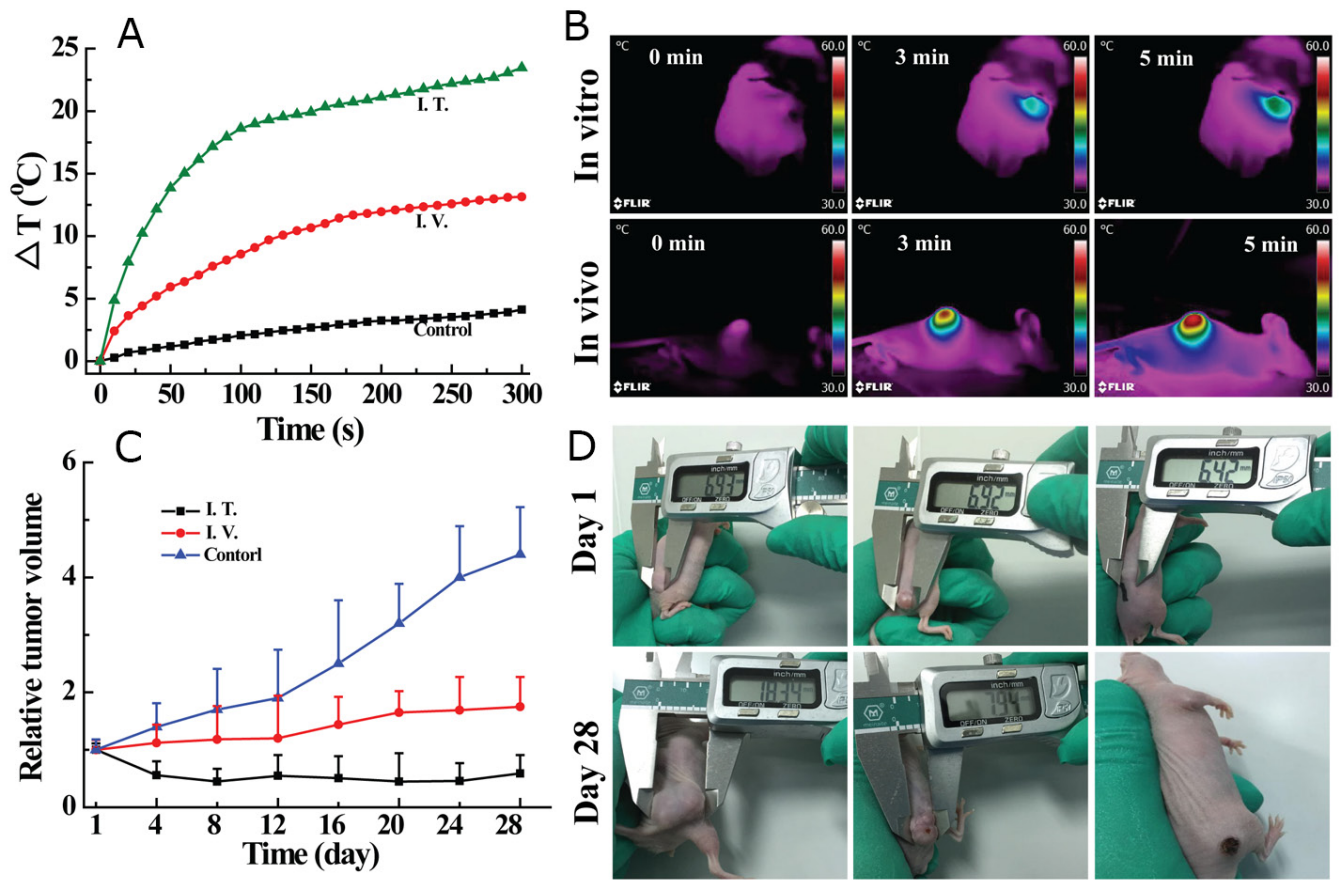
**(A)** Tumor temperature changes under NIR laser irradiation at a power density of 0. 6 W/cm^2^; **(B)** thermal images of mice before and after continuous irradiation with a NIR laser (0.8 W/cm^2^) at different time points; **(C)** tumor volume changes of mice after different treatments as indicated; **(D)** digital photos of HT29 tumor-bearing mice on day 1 and day 28 after different treatments. Mice were I.V. injected with 100 μL saline without MoS_2_-PVP nanosheets (control, left), or I.V. (center) or I.T. (right) injected with MoS_2_-PVP nanosheets.

Due to the apparent tumor retention of the I.T.-injected materials, swift tumor temperature increases of about 18.6 °C and 23.5 °C were detected after 100 and 300 s of laser irradiation, respectively (Figure 8A). The temperature increases were less remarkable for mice that were I.V. injected with MoS_2_-PVP nanosheets; moderate temperature increases of about 8.6 °C and 13.2 °C were found during 100 and 300 s of laser irradiation, respectively (Figure 8A). The ∆T distinction between the two groups was mainly due to differences in nanomaterial accumulation within the tumors; only a small portion of the nanosheets accumulated in the tumor sites of the I.V.-injected mice through the enhanced permeability retention effect. In sharp contrast, no obvious temperature fluctuation was observed in the control group; the temperature increased by only 5.6 °C, even after 300 s of laser irradiation (Figure 8A and Supplementary Figure 13).

After the tumor temperature was recorded, the mice were allowed to continue feeding so that the tumor volume could be measured over time. The tumor suppression effectiveness correlated positively with the temperature change, such that the tumor growth of I.T.-injected mice was totally inhibited (Figure 8C), and all six mice in the group remained healthy with no tumor recurrence during the entire feeding duration. It is particularly noteworthy that although the photothermal efficiency was not as obvious in the I.V.-injected group as it was in the I.T.-injected group, I.V. material injection still efficiently suppressed tumor growth. Tumor growth was significantly retarded in I.V. material-administered mice (p < 0.05, Figure 8C), with the tumor expanding to about 1.75 times its original volume after 28 days. On the other hand, uncontrolled tumor growth was observed in the control group (treated with saline + laser), such that the final tumor volumes were approximately 4.4 times larger than the initial volumes, and the occasional mouse death occurred during four weeks of feeding.

In addition to the tumor volume and survival monitoring, pathological examination by immunohistochemical staining (CD31, KI67 and TUNEL) was also performed to assess the *in vivo* tumor PTT outcomes. To determine the microvessel density, a reflection of tumor angiogenesis, we detected CD31-positive (brown-stained) cells and counted the corresponding vessels. The microvessel density was calculated as the average number of microvessels in five fields for each sample. As indicated in Supplementary Figure 14, the microvessel density decreased from 30.2 ± 1.9 (control) to 4.0 ± 1.6 (I.V., p < 0.01) and 1.4 ± 1.1 (I.T., p < 0.01). The tumor proliferation index was further determined by KI67 staining. The positive incidence percentage was 79.8 ± 2.3% in the control group, but decreased acutely to 4.6 ± 1.1% and 1.6 ± 1.3% in mice injected I.T. (p < 0.01) and I.V. (p < 0.01) with MoS_2_-PVP nanosheets, respectively. Moreover, we performed a terminal deoxynucleotidyl transferase-mediated dUTP-biotin nick end labeling (TUNEL) assay to analyze the extent of apoptosis in tumors from mice that received different treatments. The average TUNEL-positive cell levels were significantly greater in mice injected I.V. (82.5 ± 3.9%, p < 0.01) and I.T. (94.2 ± 1.64%, p < 0.01) with the nanosheets than in the control group (1.76 ± 0.83%). These immunohistochemical staining results supported the promising *in vivo* anti-cancer effectiveness of the as-synthesized MoS_2_-PVP nanosheets. Considering the strong *in vitro* and *in vivo* dual modal imaging abilities and high biocompatibility of the ultra-small MoS_2_-PVP nanosheets, we highly expect that they will have diverse applications in biomedicine.

In summary, we have reported a one-pot PVP-assisted hydrothermal route for the controlled synthesis of ultra-small MoS_2_-PVP nanosheets. Due to the chelating-coordinating effect between the lone-pair electrons of the PVP carbonyl oxygen and the unoccupied 4d orbitals of Mo, PVP was synchronously grafted onto the surface of ultra-small MoS_2_ nanosheets. PVP acted not only as a modifier to equip the MoS_2_ nanosheets with excellent colloidal stability, but also as a template to guide the growth of the nanosheets. The as-prepared MoS_2_-PVP nanosheets exhibited strong photothermal conversion efficiency and excellent cyto-, hemo- and histocompatibility that may satisfy the strict application requirements for biomedicine. Our findings not only highlight the translational potential of MoS_2_-based nanomaterials, but also may initiate further exploration into the rational design of nucleus-targeting tumor PTT systems.

## MATERIALS AND METHODS

### Materials

The precursor (NH_4_)_2_MoS_4_ was obtained from J&K Chemical Co., Ltd. (Shanghai, China). PVP (molecular weights [Mws]: 30 kDa and 360 kDa) and monoethanolamine were purchased from Sinopharm Chemical Reagent Co., Ltd. (Shanghai, China). Human colon cells (HT29) and a mouse fibroblast cell line (L929) were purchased from the Institute of Biochemistry and Cell Biology (Chinese Academy of Sciences, Shanghai, China). Roswell Park Memorial Institute 1640 (RPMI 1640) medium, Dulbecco’s Modified Eagle’s Medium (DMEM), fetal bovine serum (FBS), penicillin-streptomycin and trypan blue were bought from Gibco (Shanghai, China). Balb/c nude mice with body weights of ∼20 g and four-week-old Kunming (KM) mice were purchased from Shanghai Laboratory Animal Center (Shanghai, China). All animal experiments were carried out according to the guidelines of Changhai Hospital, Second Military Medical University and the policies of the National Ministry of Health. All chemicals were used as received. The water (with resistivity higher than 18.2 MΩ·cm) was treated in a Pall Cascada laboratory water system.

### Material preparation

Pristine MoS_2_ nanosheets were synthesized by a previously reported method [17]: 300 mg (NH_4_)_2_MoS_4_ was dissolved in 60 mL distilled water and magnetically stirred. The resultant homogenous solution was then placed in a 100-mL polyphenylene-lined stainless-steel autoclave and heated at 220 °C for 12 h. The one-pot hydrothermal synthesis of MoS_2_-PVP was performed as follows: 150 mg (NH_4_)_2_MoS_4_ and 150 mg PVP (Mw: 30 or 360 kDa) were dissolved in 30 mL water by vigorous magnetic stirring. The resultant homogenous solutions were then pipetted into 100-mL polyphenylene-lined stainless-steel autoclaves and maintained at 220 °C for 12 h. The final products were washed once with a monoethanolamine solution (50% in water, v/v) and three times with distilled water, and were stored at 4 °C for later use. The products were denoted as MoS_2_-PVP_30kDa_ and MoS_2_-PVP when the Mws of PVP were 30 kDa and 360 kDa, respectively.

### Characterization

Transmission electron microscopy (TEM) was used to observe the microtopography of MoS_2_ and MoS_2_-PVP nanosheets. The observation was performed with a JEOL-2100F analytical electron microscope under an operation voltage of 200 kV. To determine the size and thickness of the nanosheets, we randomly measured 50 sheets of each sample from the TEM image in Image J 1.40 G software (http://rsb.info.nih.gov/ij/down-load.html) [24]. The elemental composition of the MoS_2_-PVP nanosheets was analyzed with the X-ray energy dispersive spectroscopy (EDS) module of an FEI Magellan 400 field-emission microscope. The nanosheets were sprayed on mica. X-ray photo-electron spectroscopy (XPS, ESCAlab250, Thermal Scientific) was used to determine the chemical nature of the product. A Rigaku D/max-2200 PC X-ray Diffraction (XRD) system was used to study the crystalline structure of the synthesized nanosheets. The scanning parameters were a 2θ value of 3^o^ to 70^o^ and Cu Kα radiation with a wavelength of 1.54 Å operated at 40 kV and 40 mA. The surface compositions of the MoS_2_ and MoS_2_-PVP nanosheets were evaluated via Fourier transform infrared spectroscopy with a Nicolet 7000-C spectrometer under transmission mode. The scanning was performed within the wavelength range of 400 to 4000 cm^-1^. The UV-Vis-NIR light absorption features of the MoS_2_ and MoS_2_-PVP nanosheets were compared on a UV-3600 Shimadzu UV-Vis-NIR spectrometer. A NanoBrook 90Plus Particle Size Analyzer system (Brookhaven) equipped with a standard 633-nm laser was used to measure the dynamic light scattering (DLS) size of the MoS_2_-PVP nanosheets in different media and their Zeta potential in distilled water.

### Cell culture and animal model

L929 and HT29 cells were cultured in 100-mm cell petri dishes (Corning Incorporated) that were filled with 5 mL DMEM or RPMI 1640 medium containing 10% FBS, 100 units/mL penicillin and 100 μg/mL streptomycin. The cells were continuously incubated in a humidified incubator (5% CO_2_, 37 °C) and the medium was changed every two to three days. The tumor model was established through the subcutaneous injection of 150 μL serum-free RPMI 1640 medium containing 1 × 10^6^ HT29 cells into the backs of Balb/c nude mice. Animal experiments were carried out when the tumor volume reached about ∼0.5 cm^3^.

### Photothermal conversion performance

The NIR laser (wavelength = 808 nm) used in this research originated from a high-power multimode pump laser (Shanghai Connet Fiber Optics Company). The temperature and thermal images were recorded with a FLIR^™^ A320 camera (FLIR, USA). The temperature of an individual hole of a 96-well cell culture plate was recorded to evaluate the NIR photothermal transformation efficiency of the as-prepared MoS_2_-PVP nanosheets. This plate was filled with 100-μL MoS_2_-PVP nanosheet aqueous dispersions at different concentrations and continuously irradiated by the NIR laser. Distilled water was set as the control. The mass extinction coefficient (κ) can be calculated by the Lambert-Beer law: A = κ·C·L, where A is the absorbance that can be determined from the UV-Vis-NIR curve, C is the concentration of the MoS_2_-PVP nanosheets (unit: mg/mL) and L is the optical length (L = 1 cm) of the quartz microplate.

### Cellular uptake of MoS_2_ nanosheets

The cellular uptake of the MoS_2_-PVP nanosheets was studied through inductively coupled plasma optical emission spectroscopy (ICP-OES) and Bio-TEM. Briefly, HT29 cells at a density of 5 × 10^5^ cells/well were seeded in a six-well tissue culture plate and incubated overnight to allow the thorough adherence and spreading of cells. Then, the wells were replenished with RPMI 1640 medium containing MoS_2_-PVP nanosheets (200 μg/mL) and the cells were cultured for an additional 6 h. Subsequently, the cell culture medium was discarded and the cells were rinsed with phosphate-buffered saline three times. These cells were finally dissolved in 1 mL aqua regia solution for ICP-OES analysis to quantify the Mo content internalized by the cells.

Bio-TEM was performed to further assess the cellular uptake of the MoS_2_-PVP nanosheets. Approximately 8 × 10^6^ HT29 cells were seeded in a 100-mm cell culture dish and incubated overnight to allow cellular adherence and spreading. Then, the wells were replenished with RPMI 1640 medium containing MoS_2_-PVP nanosheets (1 mg/mL) and the cells were cultured for an additional 6 h. After the cell culture medium was discarded and the dish was washed with phosphate-buffered saline three times, the cells were trypsinized and fixed with 2.5 wt.% glutaraldehyde at 4 °C. Cells were visualized with a Hitachi H600 transmission electron microscope at an operating voltage of 60 kV.

### *In vitro* cytocompatibility evaluation and PTT

A typical Cell Counting Kit-8 (CCK-8) assay was used to study the *in vitro* cytocompatibility of the MoS_2_-PVP nanosheets. In brief, L929 and HT29 cells at a seeding density of ∼8 × 10^3^ cells/well were dispersed in 100 μL of the corresponding medium and added to individual wells of a 96-well plate. The cells were then incubated with fresh medium containing a pre-determined concentration of MoS_2_ nanosheets. After 24 h of incubation, the cellular metabolic activity was determined with the CCK-8 kit according to the manufacturer’s instructions. The cellular morphology was also observed with a Leica DM IL LED inverted-phase contrast microscope to evaluate the viability of the L929 and HT29 cells qualitatively.

For the *in vitro* PTT, ∼8 × 10^3^ HT29 cells dispersed in 100 μL RPMI 1640 medium were pipetted into individual wells of a 96-well plate and cultured overnight. Then, pre-determined concentrations of MoS_2_-PVP nanosheets were added and incubated for another 6 h. After that, the cells were irradiated with the NIR laser (0.6 W/cm^2^) for 1 min or 5 min and incubated for 24 h. A CCK-8 kit, Leica DM IL LED inverted-phase contrast microscope and trypan blue staining were used to evaluate the *in vitro* PTT effect quantitatively and qualitatively.

### Hemocompatibility of MoS_2_ nanosheets

For *in vitro* hemocompatibility experiments, KM mice were anesthetized for heart puncturing to collect blood. Mouse red blood cells (mRBCs) were collected following centrifugation and removal of the serum, and the cells were stored in saline at 4 °C. Then, a 0.2-mL mRBC suspension was treated with 0.8 mL of a dispersion of MoS_2_-PVP nanosheets (200 μg/mL) and saline in a 1.5-mL Eppendorf tube at 37 °C for 2 h. The treated mRBCs were harvested via centrifugation (10000 rpm, 1 min) and their morphology was visualized according to the instructions for Wright’s stain.

For *in vivo* serum biochemistry and routine blood testing, 200-μL MoS_2_-PVP nanosheet dispersions (1 mg/mL) were intravenously (I.V.) administered to KM mice. On day 1, 7, 14, or 28 after the material injection, mice were anesthetized for heart puncturing to collect blood. The harvested blood was centrifuged to separate the blood serum for biochemical parameter analysis and a coagulation assay. An ACL™ 200 blood coagulation analyzer was used to assess typical coagulation parameters, including the prothrombin time (PT), activated partial thromboplastin time (APTT) and fibrinogen level (FIB), according to the instructions of a HemosIL™ kit. For routine *in vivo* blood testing, the harvested blood was stabilized by heparin. A Sysmex XS-800i automated hematology analyzer was used to record various parameters, including the levels of white blood cells (WBC), hemoglobin (Hb), platelets (PLT), red blood cells (RBC) and hematocrit (HCT), the mean corpuscular volume (MCV), the mean corpuscular hemoglobin (MCH), the mean corpuscular hemoglobin concentration (MCHC) and the red cell distribution width (RDW).

### *In vivo* biodistribution and histology examinations

After the hemocompatibility assay, KM mice were euthanized and their major organs (heart, liver, spleen, lung and kidney) were weighed and cut into two equal parts. The first part of each organ was dissolved in an aqua regia solution to quantify the amount of Mo with an Agilent 700 Series ICP-OES. The remaining part of each organ was fixed with formaldehyde and used for hematoxylin and eosin (H&E) staining to diagnose pathological changes, according to standard procedures for H&E staining. Healthy mice without material injection were used as controls.

### *In vitro* and *in vivo* photoacoustic imaging

*In vitro* photoacoustic imaging was performed with a Vevo LAZR Photoacoustic Imaging System at a wavelength of 808 nm. Before the test, aqueous MoS_2_-PVP nanosheet dispersions (15 μL) at concentrations of 25, 50 and 100 ppm were transferred into small holes in a home-made agar plate. For the *in vivo* photoacoustic imaging, two tumor-bearing Balb/c nude mice (tumor volume: ∼0.5 cm^3^) were anesthetized and injected with either 100 μL (I.V.) or 30 μL (intratumorally [I.T.]) of a MoS_2_-PVP nanosheet dispersion. Mice with I.V. saline injection were set as controls. Mice were euthanized 12 h or immediately after the I.V. or I.T. injection, and the tumors were harvested in 1.5-mL Eppendorf tubes and fixed with formaldehyde for photoacoustic imaging (Vevo LAZR Photoacoustic Imaging System).

### *In vivo* PTT

Tumor-bearing Balb/c mice were randomly divided into three groups (n = 6 per group) and injected with 100 μL saline (I.V., control, group 1), 100 μL of the MoS_2_-PVP nanosheet dispersion (I.V., group 2) or 30 μL of the MoS_2_-PVP nanosheet dispersion (I.T., group 3). Mice in groups 1 and 3 were treated with NIR irradiation (0.6 W/cm^2^, 5 min) immediately after the injection. Mice that received I.V. material injection were treated with NIR irradiation (0.6 W/cm^2^, 5 min, group 2) 12 h after the material injection to allow time for the MoS_2_-PVP nanosheets to travel through the blood vessels and reticuloendothelial system organs and reach the tumor. The temperature and thermal images were recorded with a FLIR^™^ A320 camera (FLIR, USA). The *in vivo* PTT performance was determined based on immunohistochemical analysis, the relative tumor volume (V/V_0,_ where V_0_ represents the initial subcutaneous tumor volume at day 0) and the tumor appearance.

### Statistical analysis

One-way analysis of variance was performed to evaluate the significance of the experimental data. A significance level of 0.05 was selected, and the data were marked with (^*^) for a probability value less than 0.05 (p < 0.05), (^**^) for p < 0.01, and (^***^) for p < 0.001. Unless specified, the sample size was three (n = 3).

## SUPPLEMENTARY MATERIALS FIGURES


