# Supplementary material for: Bottom-up synthesis of ultra-small molybdenum disulfide-polyvinylpyrrolidone nanosheets for imaging-guided tumor regression
Source: Oncotarget. 2017 Nov 8;8(63):106707–20. doi: 10.18632/oncotarget.22477 (PMC5739768; doi:10.18632/oncotarget.22477)
Supplement: Supplementary file 1 [file oncotarget-08-106707-s001.pdf]

Bottom-up synthesis of ultra-small molybdenum disulfide-polyvinylpyrrolidone nanosheets for imaging-guided tumor regression

SUPPLEMENTARY MATERIALS

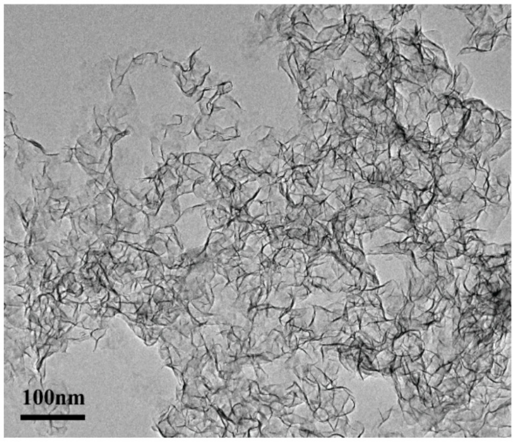

Supplementary Figure 1: TEM image of MoS_2 nanosheets.

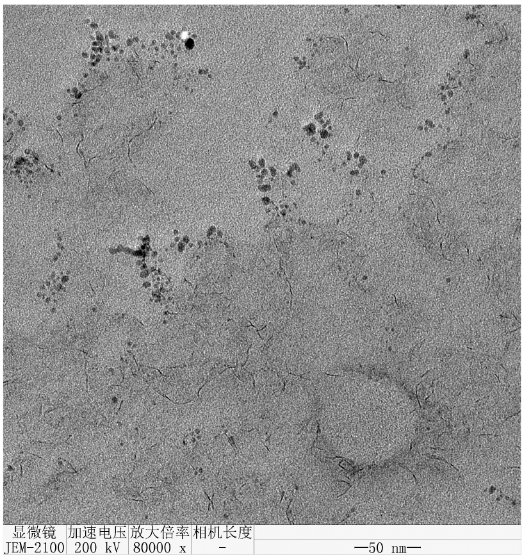

Supplementary Figure 2: TEM image of MoS_2-PVP_{30kDa} nanosheets produced when the Mw of PVP was 30kDa.

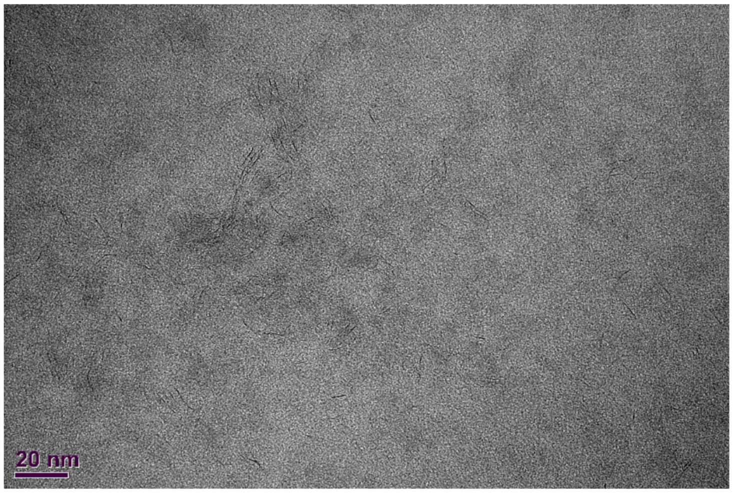

Supplementary Figure 3: TEM image of MoS_2-PVP nanosheets produced at a multiple PVP concentration (10 mg/mL).

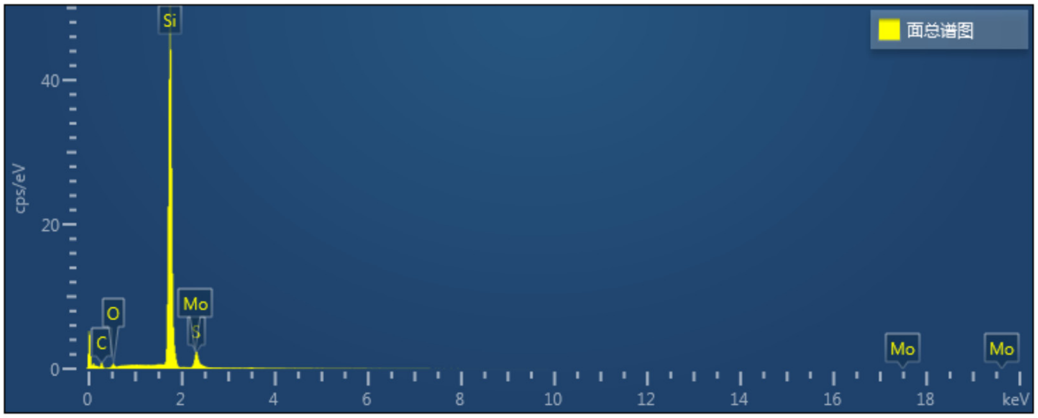

Supplementary Figure 4: X-ray energy dispersive spectroscopy (EDS) spectrum of MoS_2 nanosheets.

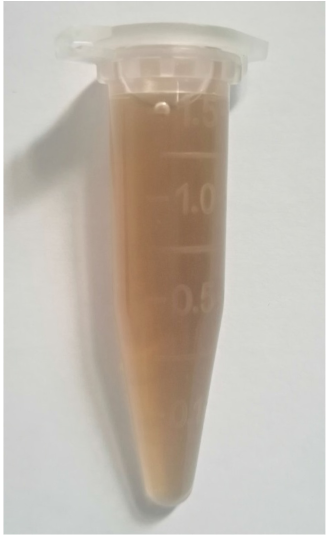

Supplementary Figure 5: Photographic image of fresh MoS_2-PVP aqueous solution (solvent: FBS).

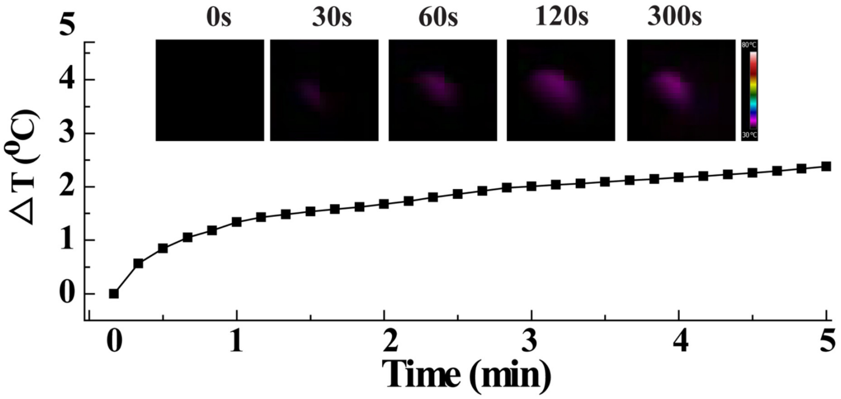

Supplementary Figure 6: Temperature profiles of water during 300 s irradiation by NIR laser at the power density of 1. 0 W/cm^2. The inserts are the corresponding thermal images at certain time points.

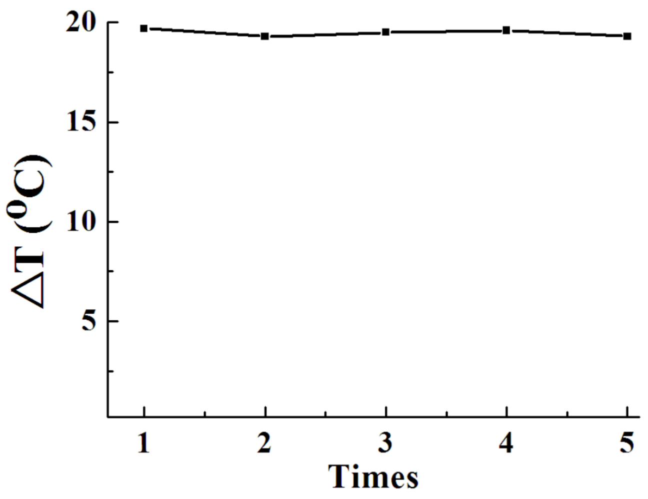

Supplementary Figure 7: Temperature variations of MoS_2 nanosheet dispersion under 5 cycles continuous irradiation of NIR laser.

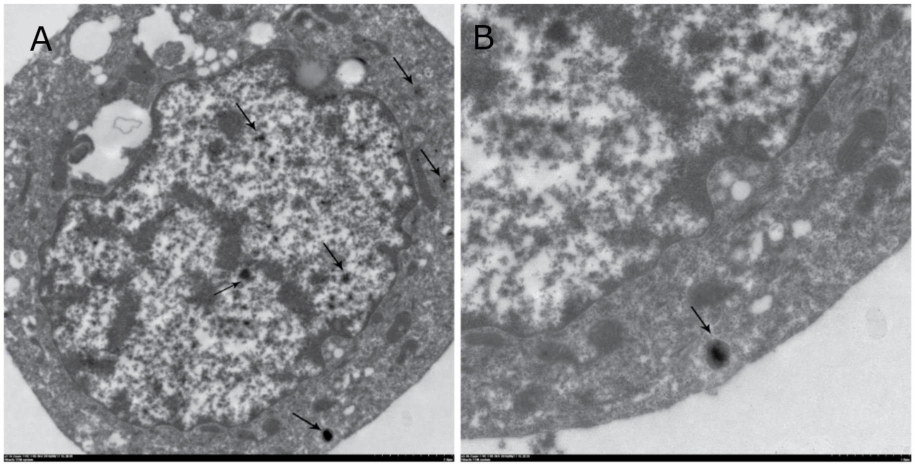

Supplementary Figure 8: Cellular uptake results: **(A)** Bio-TEM images of HT29 cells after incubated with MoS_2-PVP nanosheets (1 mg/mL) for 6 hours, MoS_2 nanosheets could be clearly detected in the cytoplasm and cytomembrane. **(B)** Enlarged image of panel a (rectangular portion). Arrows clearly show the uptake of MoS_2 nanosheets by HT29 cells.

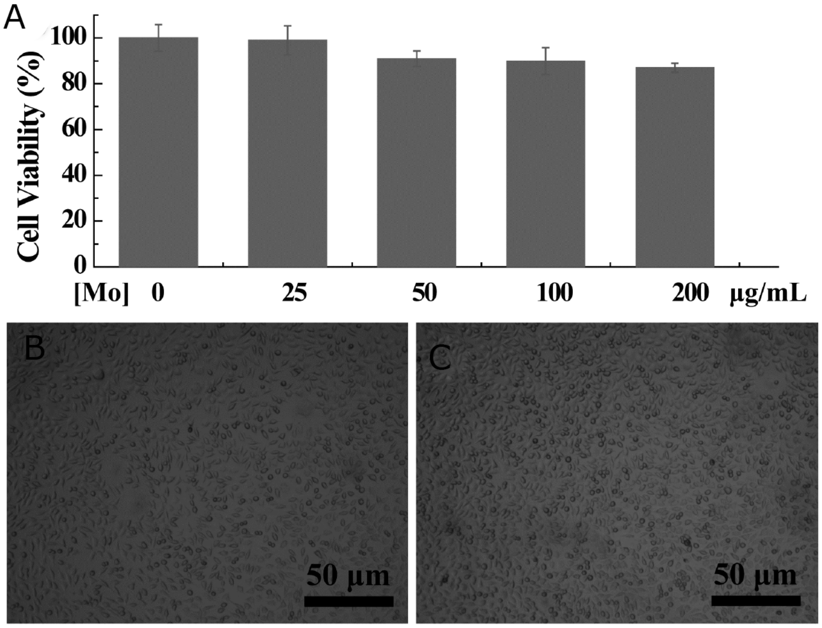

Supplementary Figure 9: (A) L929 cells viability after treated with MoS_2 nanosheets (MoS_2 nanosheets concentration: 0-200 μg/mL). **(B)** Phase-contrast L929 cell morphology Control: L929 cells treated with saline and trypan blue. **(C)** L929 cells treated with MoS_2 nanosheets at a concentration of 200 μg/mL and then stained with trypan blue.

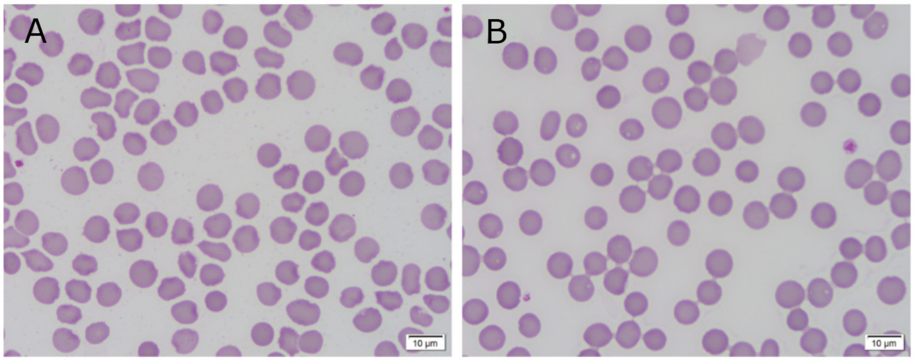

Supplementary Figure 10: Hemolytic assay results: Wright staining results show the typical morphology of mRBCs incubated with saline **(A)** or MoS_2-PVP nanosheets with a concentration of 200 μg/mL **(B)**.

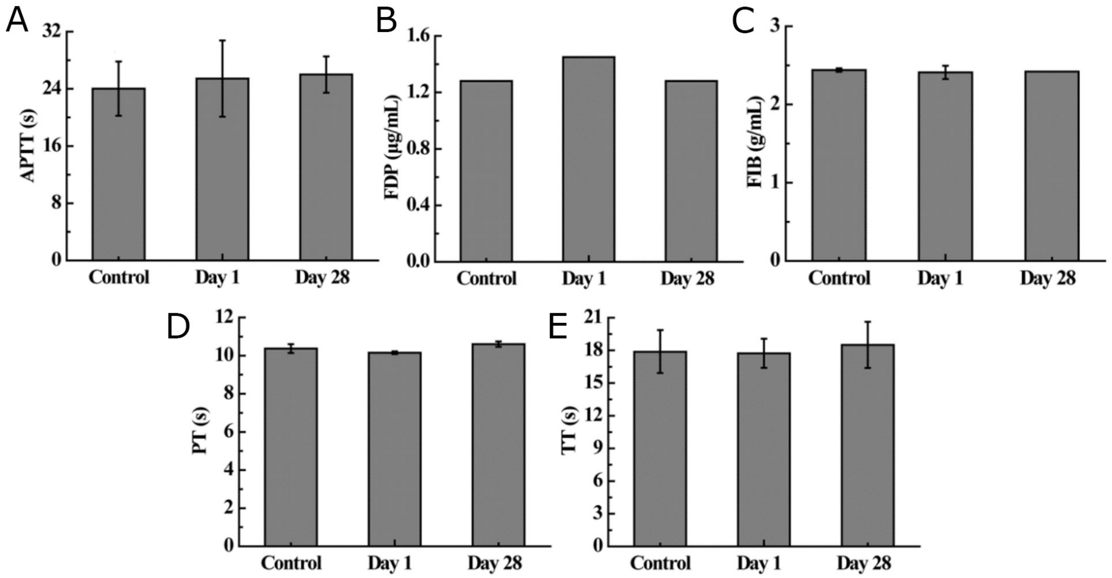

Supplementary Figure 11: *In vivo* coagulation parameters included **(A)** activated partial thromboplastin time (APTT); **(B)** fibrin(-ogen) degradation products (FDP); **(C)** fibrinogen (FIB); **(D)** prothrombin time (PT); **(E)** thrombin time (TT) of mice after I. V. injected with saline (control), or 1 and 28 days after I. V. injected with MoS2 nanosheets (mean ± SD, n = 3).

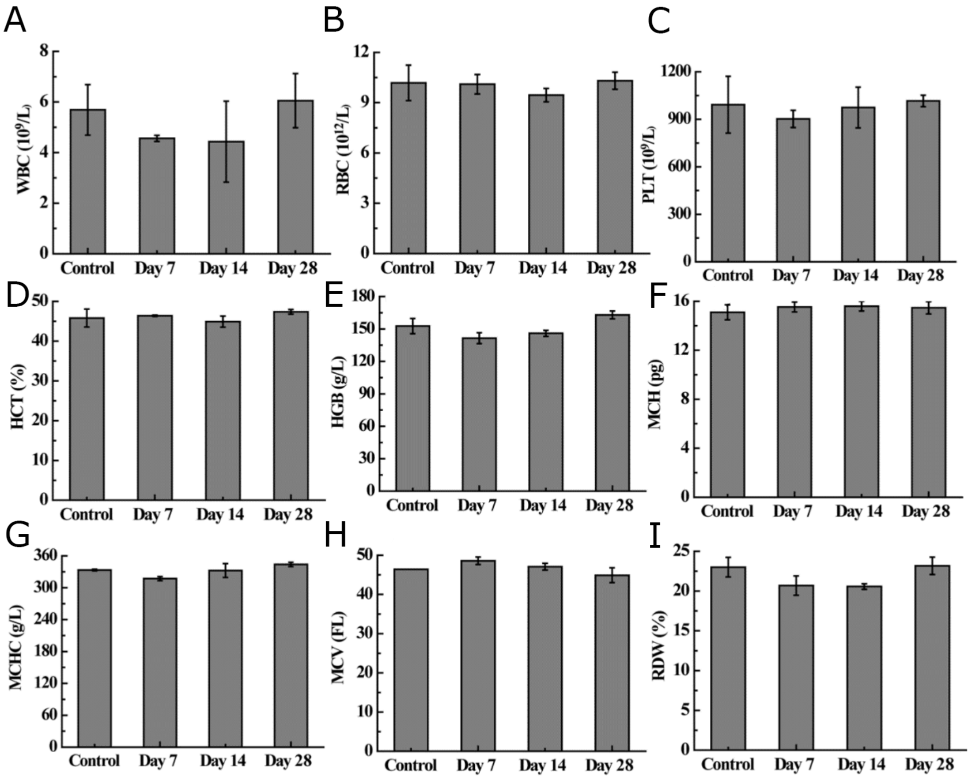

Supplementary Figure 12: Hematology data included **(A)** white blood cell count (WBC); **(B)** red blood cell count (RBC); **(C)** platelet (PLT); **(D)** hematocrit (HCT); **(E)** hemoglobin (HGB); **(F)** mean corpuscular hemoglobin (MCH);) **(G)** mean corpuscular hemoglobin concentration (MCHC); **(H)** mean corpuscular volume (MCV); **(I)** red cell distribution width (RDW) of mice after I. V. injected with saline (control), or 7, 14 and 28 days after I. V. injected with MoS2 nanosheets (mean ± SD, n = 3).

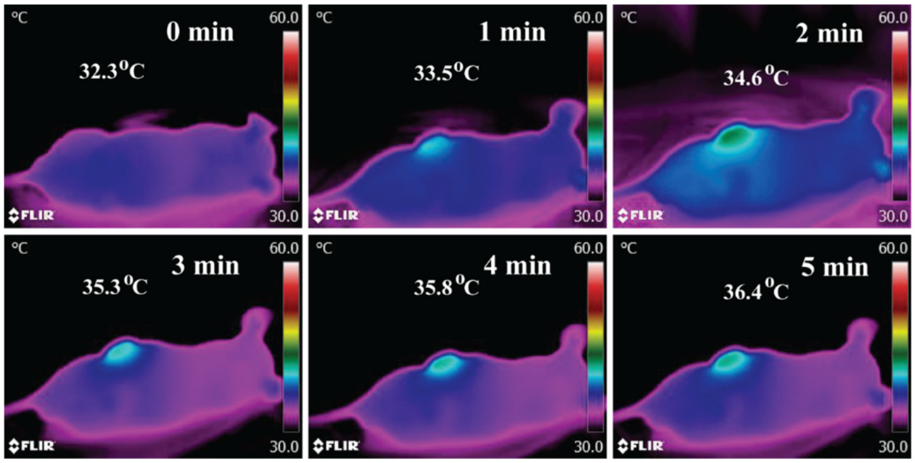

Supplementary Figure 13: Temperature profiles of mouse (I. V. injected with saline) during 300 s irradiation by NIR laser at the power density of 0.6 W/cm^2. The inserts are the corresponding thermal images at certain time points.

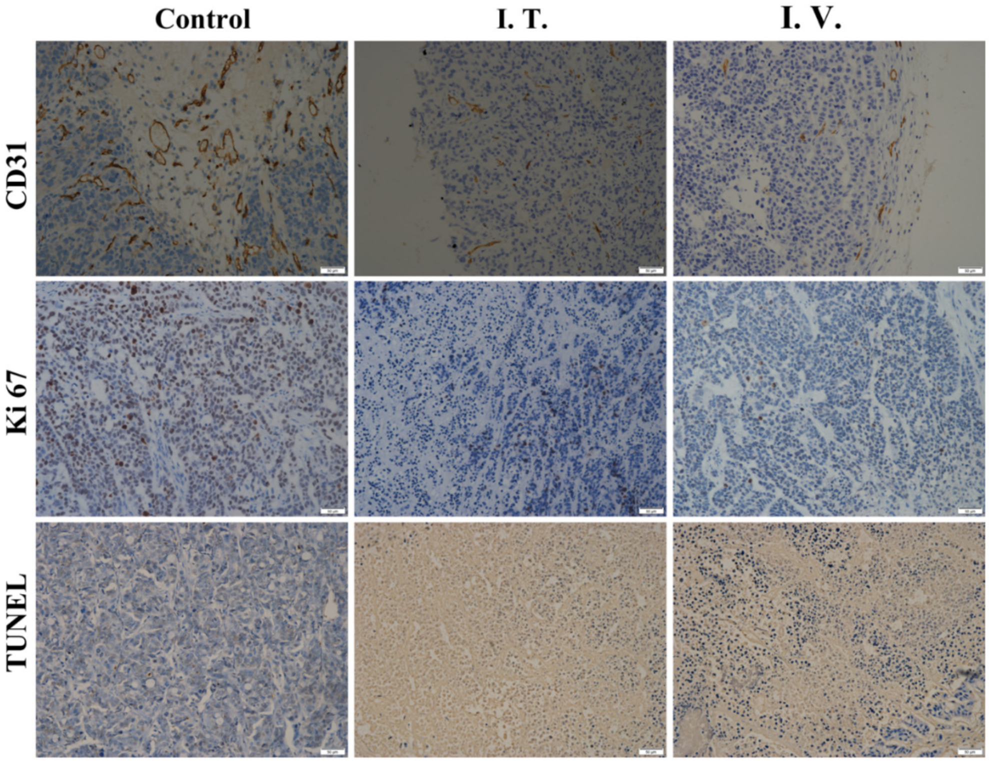

Supplementary Figure 14: Immunohistochemical staining results of CD31 expression, Ki 67 and tunel non-ablated tumor tissue (control), I. T. injection and NIR irradiated or I. V. injection and NIR irradiated tumor.
